# Genomic characterization between HER2‐positive and negative gastric cancer patients in a prospective trial

**DOI:** 10.1002/cam4.6269

**Published:** 2023-06-16

**Authors:** Qingjiang Hu, Eiji Oki, Teppei Yamada, Tomomi Kashiwada, Hideto Sonoda, Masato Kataoka, Hirofumi Kawanaka, Yasushi Tsuji, Akitaka Makiyama, Yuichiro Nakashima, Mitsuhiko Ota, Yasue Kimura, Tomoharu Yoshizumi

**Affiliations:** ^1^ Department of Surgery and Science Kyushu University Hospital Fukuoka Japan; ^2^ Department of Gastroenterological Surgery Fukuoka University Hospital Fukuoka Japan; ^3^ Department of Medical Oncology Saga Medical Center Koseikan Saga Japan; ^4^ Imari Arita Kyoritsu Hospital Nishimatsuura‐gun Japan; ^5^ Department of Surgery National Hospital Organization Nagoya Medical Center Nagoya Japan; ^6^ Clinical Research Institute / Department of Gastroenterological Surgery National Hospital Organization Beppu Medical Center Beppu Japan; ^7^ Department of Medical Oncology Tonan Hospital Sapporo Japan; ^8^ Cancer Center Gifu University Hospital Gifu Japan

**Keywords:** biomarker, gastric cancer, genomic profiling, HER2, TROX trial

## Abstract

**Background:**

We aimed to clarify the genomic characteristics of HER2‐positive and negative gastric cancer cases that potentially affect tumor progression and treatment response in a prospective trial.

**Methods:**

We collected 80 formalin‐fixed paraffin‐embedded (FFPE) samples (49 HER2+ and 31 HER2‐) from gastric cancer patients who participated in the TROX‐A1 trial (UMIN000036865). We queried a 435‐gene panel (CANCERPLEX‐JP) to generate comprehensive genomic profiling data, including the tumor mutation burden, somatic mutations, and copy number variations. In addition, the genomic differences between HER2+ and HER2‐ gastric cancer patients were analyzed.

**Results:**

Mutational analyses showed that *TP53* was the most frequently mutated gene regardless of HER2 status. *ARID1A* mutation was significantly enriched in HER2‐negative patients. The number of total mutations in HER2‐negative patients with *ARID1A* mutation was remarkably higher than that in HER2‐positive patients. Next, copy number variation analyses showed that the number of amplified genes (such as *CCNE1*, *PGAP3*, and *CDK12*) in HER2‐positive cases was significantly higher than that in HER2‐negative cases. Moreover, *PTEN* deletion was more common in HER2‐positive cases. Finally, we found that, compared with HER2‐positive patients, HER2‐negative patients tended to have a higher tumor mutation burden, particularly in patients with *ARID1A* mutation. Pathway analyses of the gene alterations showed an enrichment of several immune‐related pathways in HER2‐negative patients.

**Conclusions:**

According to the genomic profiling of HER2‐positive and negative gastric cancer, several gene alterations in the HER2 pathway may be the potential mechanism underlying trastuzumab resistance. Relative to HER2‐positive gastric cancer, HER2‐negative gastric tumors with *ARID1A* mutation may be sensitive to immune checkpoint inhibitors.

## Graphical abstract

This study clarified the genomic characteristics of HER2‐positive and negative gastric cancer, providing evidence for understanding the mechanism underlying tumor progression and treatment responses in HER2‐positive and negative patients.

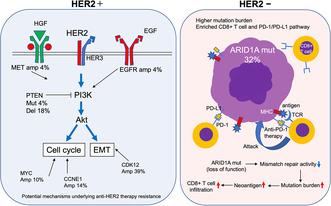

## 1 INTRODUCTION

Gastric cancer is a common malignant neoplasm and one of the leading causes of cancer‐related death worldwide.^1^ Limited targeted treatment options contribute to the poor prognosis of metastatic gastric cancer patients. Recently, precision medicine methods focused on genetic alterations have improved the prognosis of patients with solid tumors, including gastric cancer.^2^ In gastric cancer, targeted therapies have been developed and demonstrated to be effective for patients with human epidermal growth factor receptor 2 (HER2)‐positive,^3^ microsatellite instability (MSI)‐High,^4^ and neurotrophic tyrosine receptor kinase (*NTRK*) gene fusions.^5^ Recently, a phase 3 clinical trial named SPOTLIGHT showed that Zolbetuximab, an anti‐claudin 18.2 antibody, improved progression‐free survival (PFS) and overall survival (OS) in claudin 18.2‐positive, HER2‐negative gastric cancer patients (NCT03504397). Moreover, inhibitors of fibroblast growth factor receptor (*FGFR*) gene alterations are being developed in several clinical studies for gastric cancer (TAS‐120: NCT04189445, FORTUNE Trial: NCT04962867). These findings may help provide new standard therapies in the future. With the rise of next‐generation sequencing (NGS) methods for genomic profiling, both research and development approaches and clinical practice concerning targeted therapy have changed drastically.

HER2, encoded by the *ERBB2* gene, is overexpressed/amplified in approximately 15% of gastric cancer patients.^3^, ^6^, ^7^ *ERBB2* is an oncogene that drives tumor progression via activation of PI3K/Akt pathway. Thus, targeting HER2 is an attractive strategy for treating gastric cancer cases that have HER2 overexpression. In 2010, the ToGA trial results showed that the combination of trastuzumab, an anti‐HER2 antibody, and chemotherapy had a survival benefit in HER2‐positive gastric cancer patients.^3^ Median OS in the trastuzumab group was 13.8 months versus 11.1 months in the chemotherapy‐only group (hazard ratio (HR) = 0.74; 95% confidence interval (CI) = 0.60–0.91; *p* = 0.0046). These results established trastuzumab and chemotherapy as the first‐line therapy for patients with HER2‐positive gastric cancer. However, some cases of HER2‐positive gastric cancer did not benefit from trastuzumab therapy. Several previous studies presented immunohistochemistry (IHC) data that showed that low phosphatase and tensin homolog (PTEN) expression is a reason for trastuzumab resistance in gastric cancer.^8^, ^9^, ^10^ To further clarify the mechanism underlying anti‐HER2 therapy resistance, comprehensive genomic analyses of HER2‐positive gastric cancer cases using NGS may be an efficient approach.

We previously identified several novel driver genes involved in gastric cancer progression through comprehensive genomic analyses.^11^, ^12^, ^13^ In this study, we evaluated the genomic profiles of 80 gastric cancer patients using a large gene panel based on NGS analyses who participated in the TROX‐A1 trial (UMIN000036865). We compared the genomic profiles of 49 HER2‐positive and 31 HER2‐negative gastric cancer patients and then examined the potential influences of the genomic differences on tumor progression and treatment responses, including the mechanism underlying anti‐HER2 therapy resistance in gastric cancer patients.

## 2 MATERIALS AND METHODS

### 2.1 Patients and sample collection

This was a companion study to the TROX trial, preplanned as the TROX‐A1 trial (UMIN000036865). HER2‐positive patients were enrolled in the parent study (TROX trial), a randomized, open‐label, multicenter, phase II trial comparing regimens of trastuzumab biosimilar combined with oxaliplatin, either plus TS‐1 or capecitabine, as a first‐line therapy for HER2‐positive metastatic gastric cancer patients (jRCTs071190007). The key inclusion criteria of the TROX trial were as follows: (I) the individual provided written informed consent for receiving the protocol treatment; (II) the patient is 20–80 years old; (III) patients with gastric adenocarcinoma confirmed by histopathology; (IV) patients with advanced/recurrent gastric cancer not amenable to curative surgery; (V) patients with no prior antitumor therapy, including chemotherapy, radiotherapy, targeted therapy, or immunotherapy; (VI) patients with the Eastern Cooperative Oncology Group (ECOG) performance status score of ≤1. HER2‐negative gastric cancer patients were enrolled as controls in the TROX‐A1 trial with the same inclusion criteria as the TROX trial.

The HER2 status of each patient was determined by clinicians using IHC and/or fluorescence in situ hybridization (FISH). HER2‐positive was defined as an IHC score of 3 or an IHC score of 2 with a FISH HER2/CEP17 score ≥2. We collected formalin‐fixed, paraffin‐embedded sections (FFPE) from 49 HER2‐positive and 31 HER2‐negative gastric cancer patients and extracted genomic DNA from the FFPE sections.

### 2.2 Genomic DNA extraction

Genomic DNA was extracted from FFPE sections using methods previously described.^14^ For quality control (QC) purposes, extracted genomic DNA was evaluated by measuring DIN with TapeStation (Agilent Technologies). Only samples with genomic DNA quantitation >50 ng or 20–50 ng with DIN >3.5 were used for NGS analyses.

### 2.3 Genomic profiling by NGS

We evaluated the genomic profiles of 80 gastric cancer patients using the CANCERPLEX‐JP panel (DENKA Kew Genomics), as previously described,^15^ which examines 435 genes detecting single nucleotide variants (SNVs), indels, copy number variants (CNVs), and fusions. In addition, the tumor mutational burden (TMB) was determined using the algorithm of the CANCERPLEX‐JP panel.

### 2.4 The Cancer Genome Atlas

We obtained gene expression data (RNA‐seq) from 415 gastric cancer samples, CNV data from 441 gastric cancer samples, and somatic mutation data from 395 gastric cancer samples in the Firehose pipeline at the Broad Institute (http://firebrowse.org/?cohort=STAD). The mRNA expression data (FPKM values, raw counts) were subjected to quantile normalization as previously described.^11^ We calculated the (cytolytic activity) CYT score using the geometric mean of *GZMA* and *PRF1* mRNA expression levels in the The Cancer Genome Atlas (TCGA) dataset as previously described.^16^ Patients with *ERBB2* copy number >2.5 was defined as *ERBB2* amplification. Total mutation numbers in gastric cancer tumor tissues were counted according to somatic mutation data.

### 2.5 Database for annotation, visualization, and integrated discovery

We used the Database for annotation, visualization, and integrated discovery (DAVID) online tool (https://david.ncifcrf.gov) for pathway analyses of gene alterations based on HER2 status. The input genes, identified as the gene alterations enriched in HER2‐positive and HER2‐negative gastric cancer patients, are listed in Table S1. The significance of enrichment is expressed as a q‐value in the DAVID online tool. The *q*‐value is a false discovery rate (FDR) adjusted *p*‐value, with <0.05 considered significant in this study.

### 2.6 Statistical analysis

Statistical analyses with the Fisher's exact test, Student's *t*‐test, Mann–Whitney *U* test, and visualization were performed using R version 3.3.2 (R Foundation) and JMP Pro 15 software (SAS Institute). A two‐sided *p*‐value <0.05 was considered significant in this study. FDR adjustment (Benjamini–Hochberg method) was performed in pathway analyses (multiple testing). A *q*‐value <0.05 was considered significant. In the Fisher's exact test, the odds ratio (OR) was calculated, with OR >2 or <1/2 defined as an enriched gene alteration.

## 3 RESULTS

### 3.1 Genomic profiling by NGS using 80 gastric cancer tissues

**FIGURE 1:**
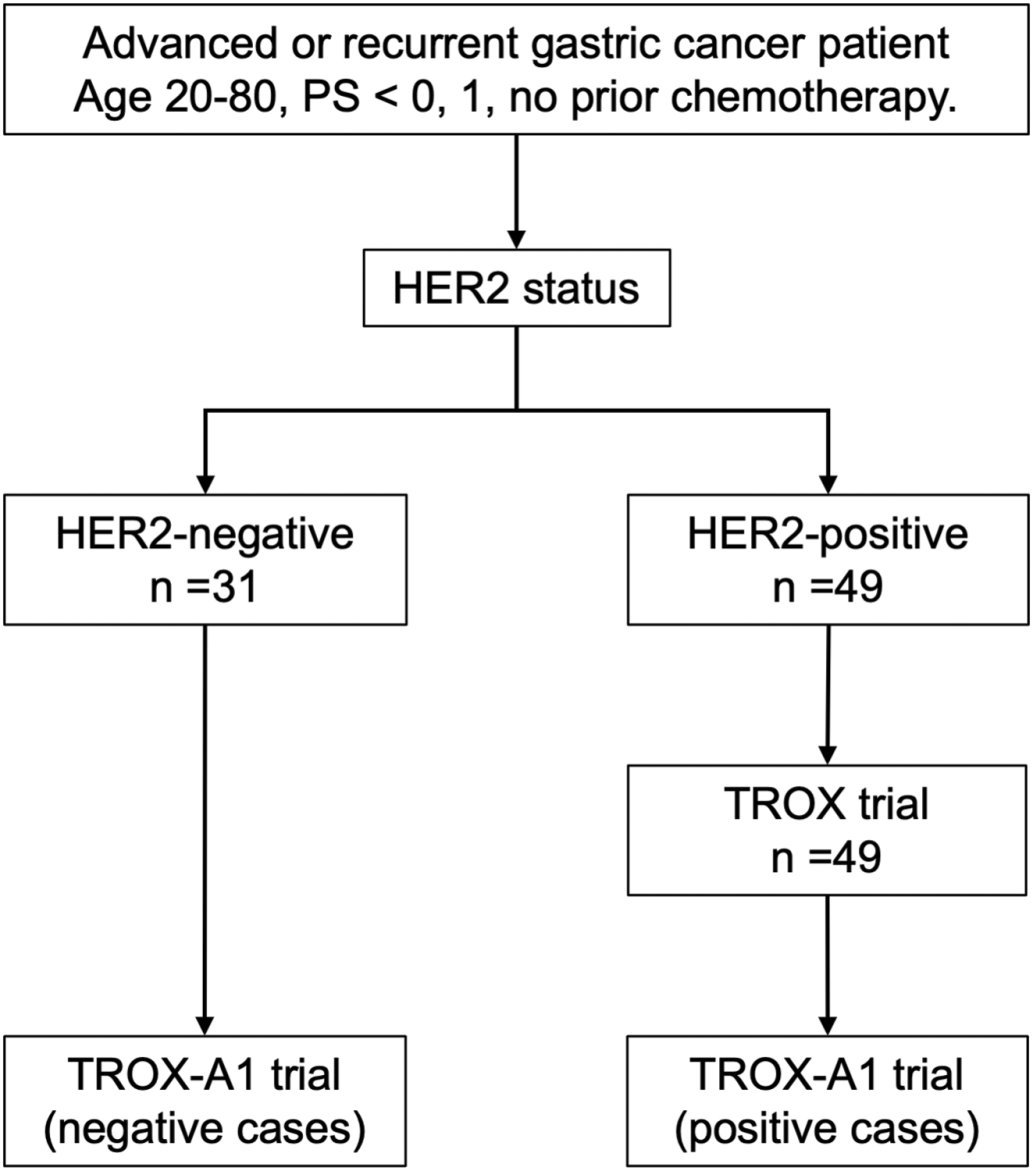
Study flowchart of the TROX‐A1 trial (*n* = 80).

**FIGURE 2:**
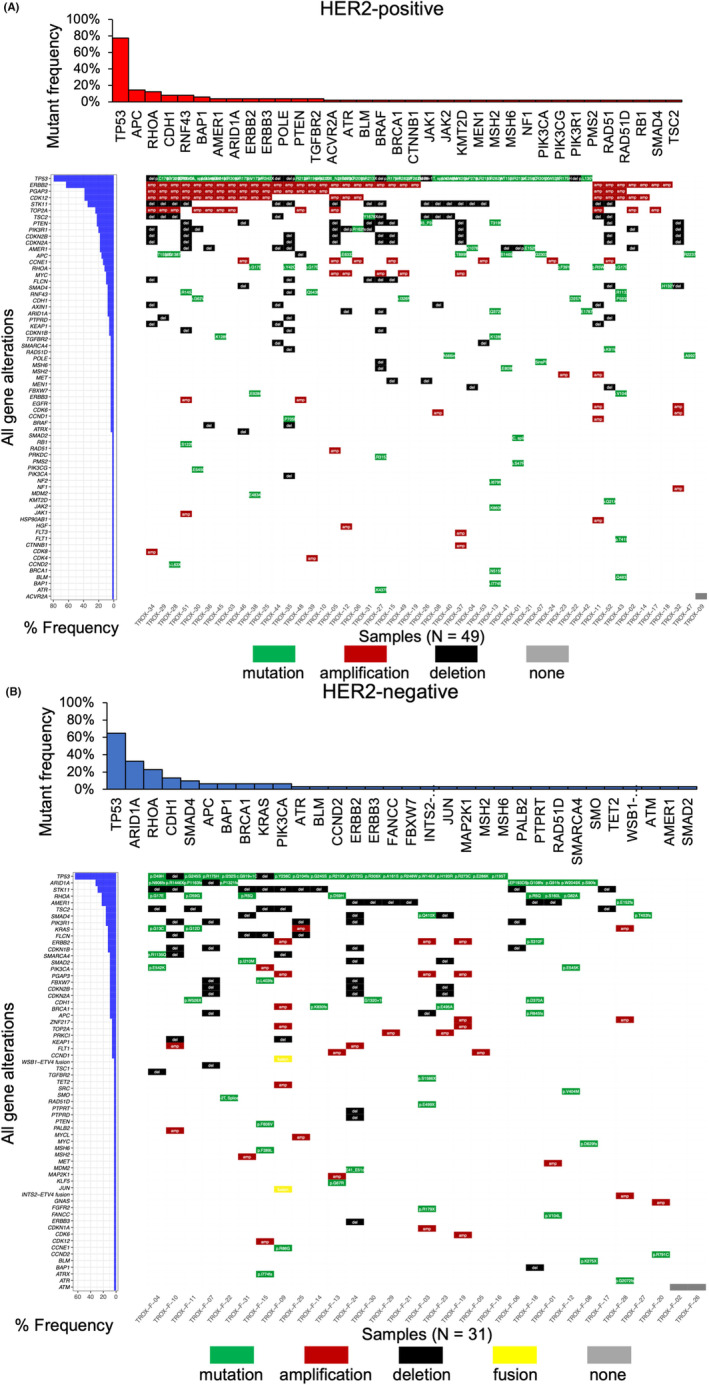
Genomic landscape based on HER2 status in this study (*n* = 80). Middle panel: All gene alterations identified by CANCERPLEX‐JP are shown in the tile plots. Green represents a mutation with detailed data described in each box; red represents amplification; black represents a deletion; yellow represents a fusion gene; gray represents no gene alterations found. Left panel: Bar plots denote the individual prevalence of all gene alterations. Upper panel: Bar plots denote the individual prevalence of all gene mutations. (A) Molecular characteristics of HER2‐positive gastric cancer patients (*n* = 49). (B) Molecular characteristics of HER2‐negative gastric cancer patients (*n* = 31).

We enrolled 49 HER2‐positive and 31 HER2‐negative gastric cancer patients in this study (TROX‐A1 trial, Figure 1). The background data of all patients are shown in Table S2. Using the CANCERPLEX‐JP reports, we generated comprehensive genomic profiles of the 80 gastric cancer patients. Ninety‐eight nonsynonymous mutations, 107 amplifications, and 114 deletions were identified in the 49 HER2‐positive gastric cancer patients. Seventy‐four nonsynonymous mutations, 31 amplifications, and 61 deletions were identified in the 31 patients with HER2‐negative gastric cancer. We explored the genomic landscape within the HER2‐defined subgroups (Figure 2A,B). As shown in Figure 2, the most frequently somatically mutated genes were *TP53* (78%), *APC* (14%), and *RHOA* (12%), and the most frequent CNVs were *ERBB2* amplification (63%), *CDK12* amplification (39%), and *PGAP3* amplification (39%) in HER2‐positive gastric cancer patients. In HER2‐negative patients, *TP53* mutation (65%), *ARID1A* mutation (32%), *RHOA* mutation (23%), *STK11* deletion (29%), *AMER1* deletion (19%), and *TSC2* deletion (16%) were the most frequent gene alterations.

### 3.2 Nonsynonymous mutations between HER2‐positive and HER2‐negative gastric cancer patients

**FIGURE 3:**
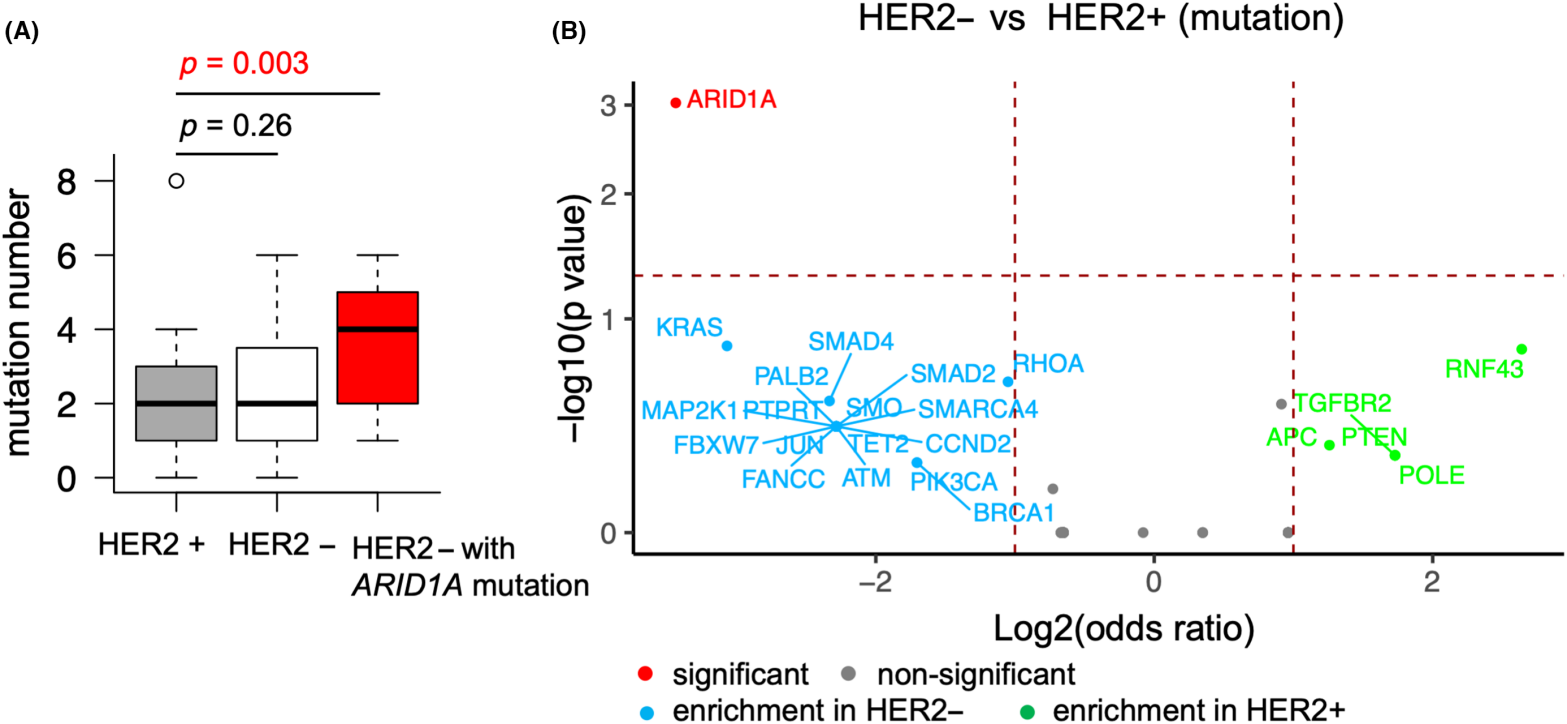
Differences in nonsynonymous mutations between HER2‐positive and HER2‐negative gastric cancer. HER2+, HER2‐positive; HER2‐, HER2‐negative. (A) The numbers of mutations in HER2‐positive patients (*n* = 49), HER2‐negative patients (*n* = 31), and HER2‐negative patients with *ARID1A* mutation (*n* = 10) are shown in the boxplot. Mann–Whitney *U* test *p*‐value <0.05 is shown in red. (B) Volcano plot showing the enriched gene mutations in HER2‐positive (*n* = 49) and HER2‐negative (*n* = 31) gastric cancer patients. The horizontal madder red line denotes a *p*‐value of 0.05, and the two vertical madder red lines denote a log2 odds ratio (OR) of 1. Only the genes that have log2 OR >1 are annotated. Fisher's exact tests were performed, and the genes with *p* < 0.05 are annotated in red.

Next, we compared nonsynonymous mutations between HER2‐positive and HER2‐negative gastric cancer cases. There were no significant differences in the number of nonsynonymous mutations between HER2‐positive and HER2‐negative patients (Figure 3A). However, the total mutational numbers in HER2‐negative patients with *ARID1A* mutation were significantly higher than in HER2‐positive patients (Figure 3A, Mann–Whitney *U* test *p* = 0.003). The median number of nonsynonymous mutations in HER2‐negative tumors with *ARID1A* mutation and HER2‐positive tumors with *ARID1A* mutation was 4 and 5.5, respectively (Figure S1). The volcano plot shows the enrichment of nonsynonymous mutations based on the HER2 status (Figure 3B). *ARID1A* mutation was significantly enriched in HER2‐negative patients (Fisher's exact test *p* < 0.001). Although not significant, the enriched mutations in HER2‐negative patients included *KRAS*, *RHOA*, *SMAD4*, *FBXW7*, *SMO*, *ATM*, *MAP2K1*, *JUN*, *PTPRT*, *SMARCA4*, *TET2*, *FANCC*, *CCND2*, *PALB2*, *BRCA1*, *SMAD2*, and *PI3KCA*. The enriched mutations in HER2‐positive patients were *RNF43*, *APC*, *PTEN*, *POLE*, and *TGFBR2*.

### 3.3 CNVs between HER2‐positive and HER2‐negative gastric cancer patients

**FIGURE 4:**
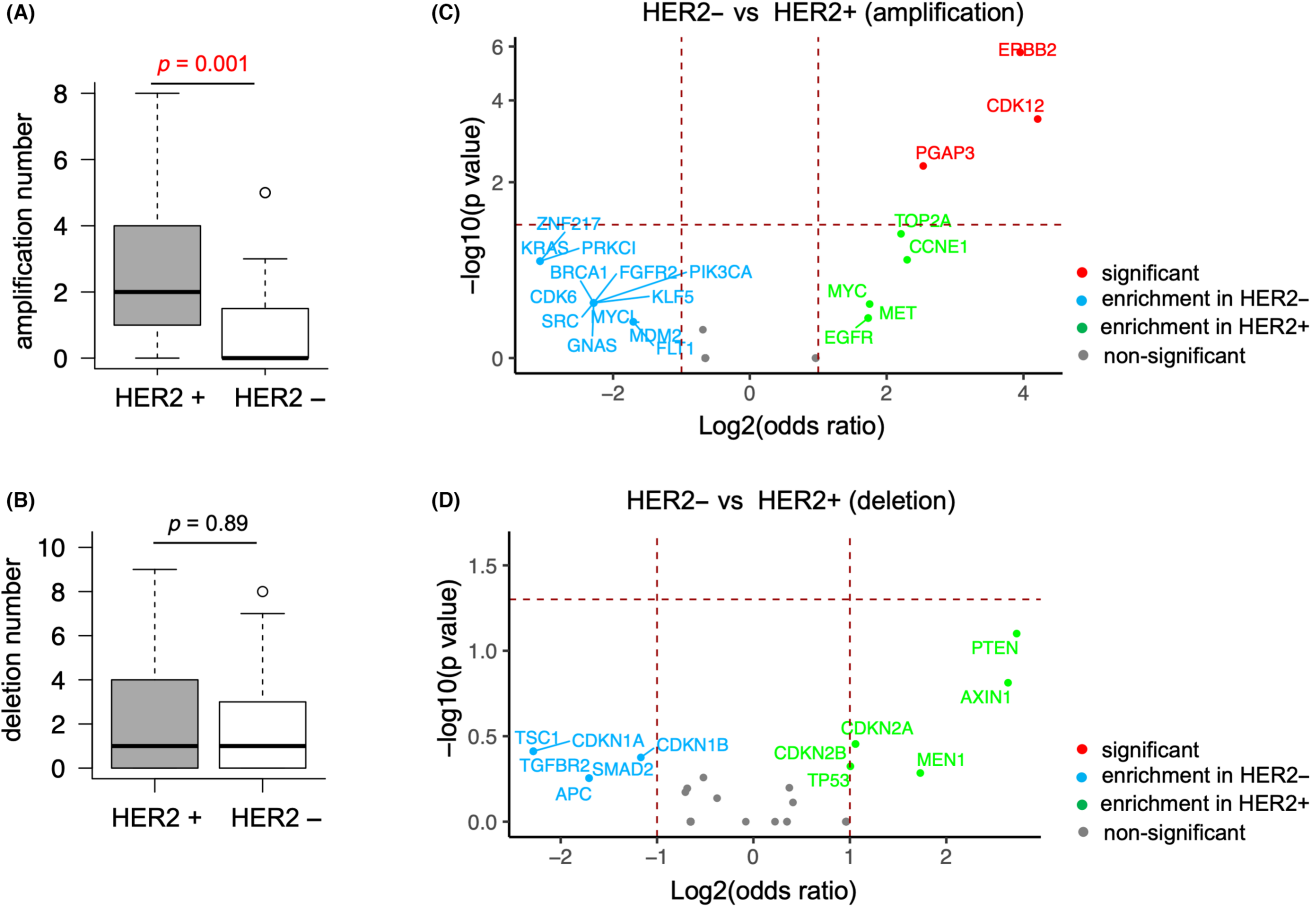
Differences in copy number variations (CNVs) between HER2‐positive and HER2‐negative gastric cancer patients. HER2+, HER2‐positive; HER2‐, HER2‐negative. The horizontal madder red line denotes a *p*‐value of 0.05, and the two vertical madder red lines denote a log2 odds ratio (OR) of 1. Only the genes that have log2 OR >1 or < −1 are annotated. Fisher's exact tests were performed, and the genes with *p* < 0.05 are annotated in red. (A) The numbers of amplifications in HER2‐positive patients (*n* = 49) and HER2‐negative patients (*n* = 31) are shown in the boxplot. Mann–Whitney *U* test *p*‐value <0.05 is shown in red. (B). The numbers of deletions in HER2‐positive patients (*n* = 49) and HER2‐negative patients (*n* = 31) are shown in the boxplot. (C) Volcano plot showing the enriched gene amplifications in HER2‐positive and HER2‐negative patients. (D) Volcano plot showing the enriched gene deletions in HER2‐positive and HER2‐negative patients.

Furthermore, we compared the gene alterations of CNVs between HER2‐positive and HER2‐negative gastric cancer cases. The number of gene amplifications was significantly higher in HER2‐positive patients than in HER2‐negative patients (Figure 4A, Mann–Whitney *U* test *p* = 0.001). There were no significant differences in the number of gene deletions between HER2‐positive and HER2‐negative patients (Figure 4B). Additionally, the amplifications of *ERBB2*, *CDK12*, and *PGAP3* were significantly enriched in HER2‐positive patients (Figure 4C, Fisher's exact test *p* < 0.01). Although not significantly, the amplifications of *TOP2A*, *CCNE1*, *MYC*, *MET*, and *EGFR*, and the deletions of *PTEN*, *AXIN1*, *CDKN2A*, *CDKN2B*, *MEN1*, and *TP53* were enriched in HER2‐positive patients (Figure 4C,D). In HER2‐negative patients, the enriched CNVs included amplifications of *PRKCI*, *ZNF217*, *KRAS*, *PIK3CA*, *CDK6*, *GNAS*, *FGFR2*, *MYCL*, *FLT1*, *KLF5*, *SRC*, *BRCA1*, *MDM2*, and *FLT1*, and deletions of *TSC1*, *CDKN1A*, *TGFBR2*, *APC*, *SMAD2*, and *CDKN1B* (Figure 4C,D).

### 3.4 Pathway analyses using gene alterations

**FIGURE 5:**
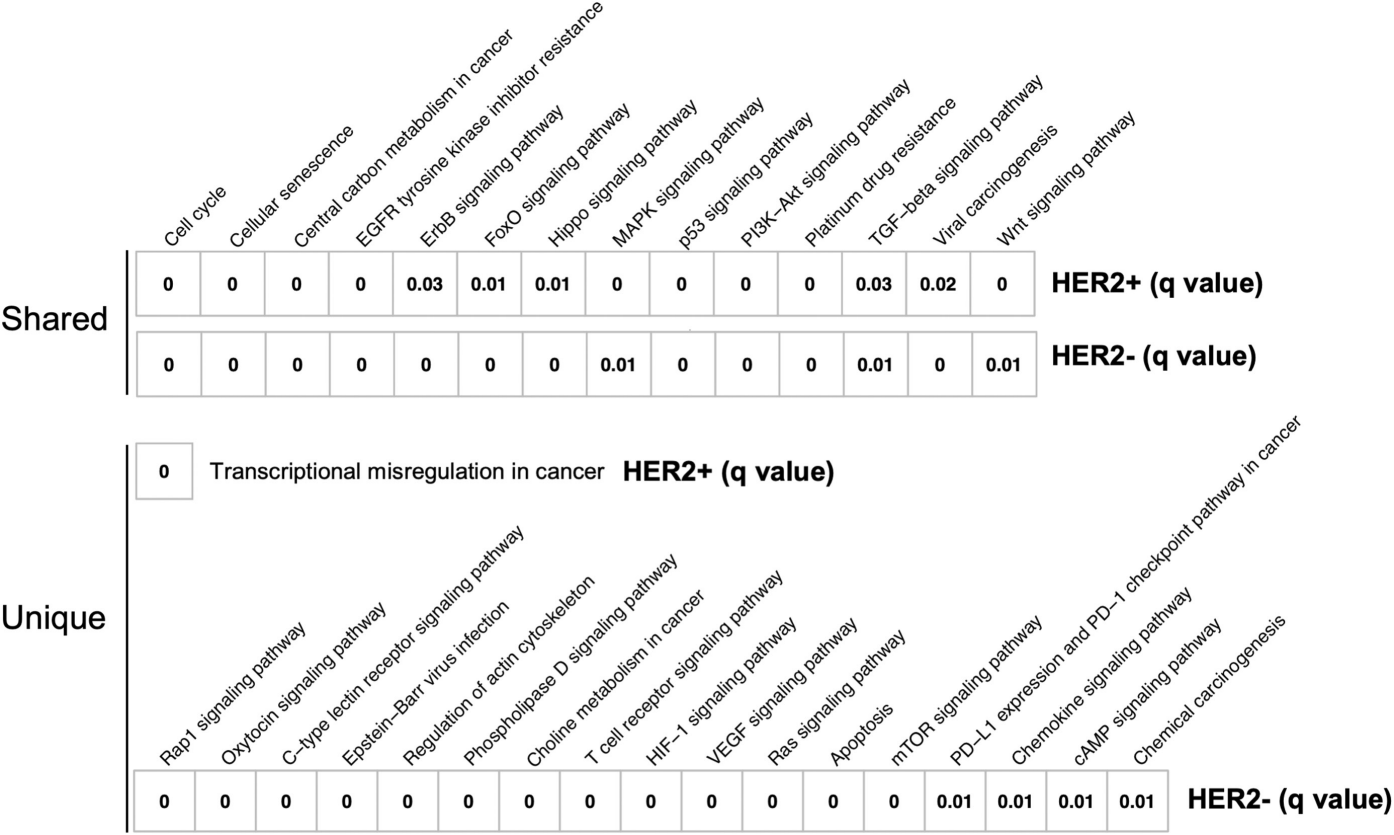
Pathway analyses for the enriched gene alterations in HER2‐positive and HER2‐negative gastric cancer. The FDR‐adjusted *p*‐value (*q*‐value) is shown in each box and represents the significance of associations between KEGG pathways and HER2 status. The pathways significantly enriched in both HER2‐positive and negative patients are defined as shared pathways. The pathways only significantly enriched in HER2‐positive or negative patients are defined as unique pathways.

According to the enriched gene alterations, we performed pathway analyses to clarify the potential mechanism underlying tumor progression in HER2‐positive and negative gastric cancer patients. We assessed the enrichment of the KEGG pathway using DAVID bioinformatics resources, as described in the Materials and Methods section. The pathways significantly enriched in both HER2‐positive and negative patients were defined as shared pathways, while those only significantly enriched in HER2‐positive or negative patients were defined as unique pathways. As shown in Figure 5, 14 shared pathways were identified, most of which were critical for tumorigenesis and progression. Only one pathway, transcriptional misregulation in cancer, was a unique pathway of HER2‐positive patients. Seventeen unique pathways of HER2‐negative patients were identified, including several immune‐related pathways.

### 3.5 Comparison of TMB between HER2‐positive and HER2‐negative gastric cancer patients

**FIGURE 6:**
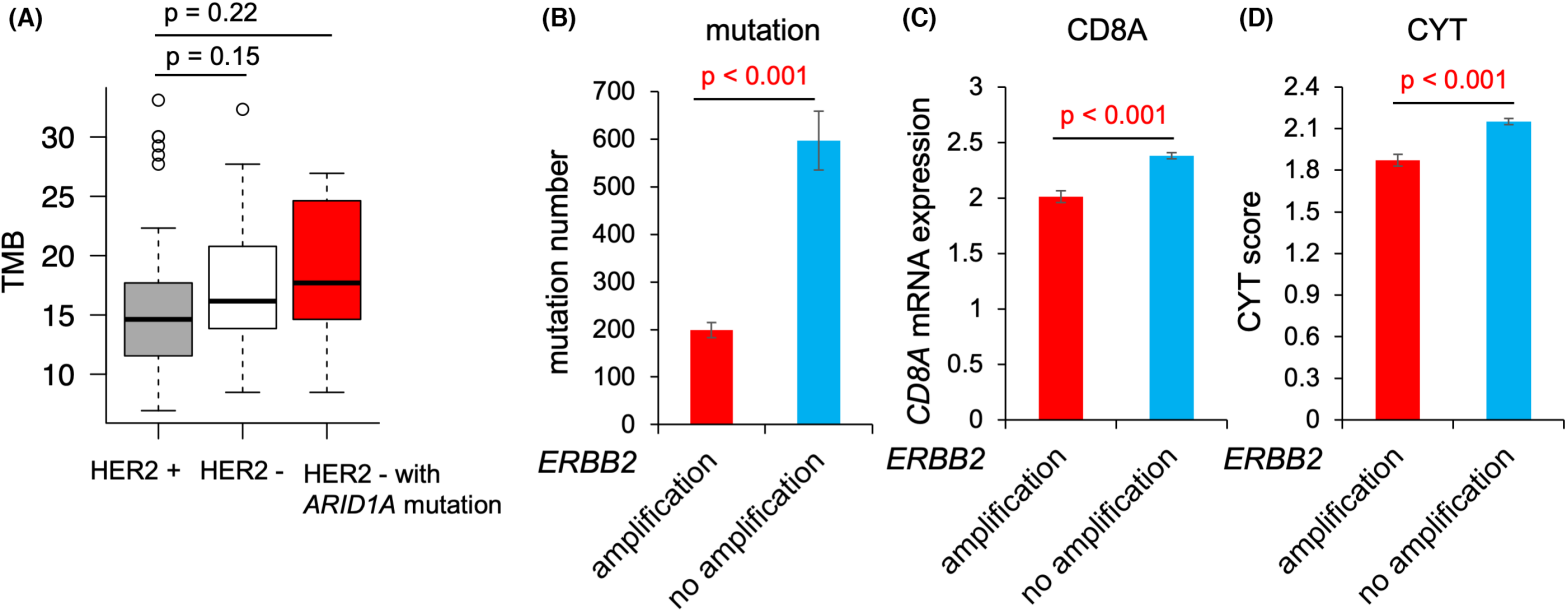
Associations between HER2 status and local immune environment factors. (A) TMB of HER2‐positive patients (*n* = 49), HER2‐negative patients (*n* = 31), and HER2‐negative patients with *ARID1A* mutation (*n* = 10) are shown in the boxplot. (B) Bar plot showing the total number of mutations in *ERBB2*‐amplified gastric cancer patients (*n* = 70) and *ERBB2*‐nonamplified gastric cancer patients (*n* = 323) from The Cancer Genome Atlas (TCGA) dataset. Student's *t*‐test *p*‐value <0.05 is shown in red. (C) Bar plot showing *CD8A* mRNA expression levels in *ERBB2*‐amplified gastric cancer patients (*n* = 75) and *ERBB2*‐nonamplified gastric cancer patients (*n* = 338) from the TCGA dataset. Student's *t*‐test *p*‐value <0.05 is shown in red. (D) Bar plot showing the CYT score in *ERBB2*‐amplified gastric cancer patients (*n* = 75) and *ERBB2*‐nonamplified gastric cancer patients (*n* = 338) from the TCGA dataset. Student's *t*‐test *p*‐value <0.05 is shown in red.

In addition, we focused on TMB differences between HER2‐positive and HER2‐negative gastric cancer patients. TMB, defined as the rate of peptide‐changing SNVs per Mb, was determined using the CANCERPLEX‐JP panel. The median TMB value for HER2‐positive patients, HER2‐negative patients, and HER2‐negative patients with *ARID1A* mutation was 14.6, 16.2, and 17.7, respectively (Figure 6A, Mann–Whitney *U* test, HER2‐positive vs HER2‐negative *p* = 0.15 & HER2‐positive vs HER2‐negative with *ARID1A* mutation *p* = 0.22). The median TMB value for HER2‐positive patients with *ARID1A* mutation was 16.2 (Figure S1). There was a trend toward a higher TMB in HER2‐negative gastric cancer patients, particularly in patients with *ARID1A* mutation, though this was not statistically significant.

### 3.6 Association between the local immune environment and *ERBB2* amplification in gastric cancer patients

We also assessed the associations between *ERBB2* amplification and local immune environment factors in gastric cancer patients from the TCGA dataset. Local immune environment factors included the number of nonsynonymous mutations, *CD8A* mRNA expression levels, and CYT score, which has already been reported as a quantitative method of antitumor immunity.^16^ The total number of nonsynonymous mutations was significantly higher in patients without *ERBB2* amplification (*n* = 323) compared with that in patients with *ERBB2* amplification (*n* = 70; Figure 6B, Student's *t*‐test *p* < 0.001). Moreover, *CD8A* mRNA expression levels, a marker of CD8+ T cells, and CYT score were both significantly higher in patients without *ERBB2* amplification (*n* = 338) than in patients with *ERBB2* amplification (*n* = 75; Figure 6C,D, Student's *t*‐test *p* < 0.001).

### 3.7 HER2/ *ERBB2* concordance in this study

Lastly, we assessed the concordance between HER2 status determined by clinicians using traditional methods and *ERBB2* amplification measured by the CANCERPLEX‐JP panel (Table S3). HER2 positivity was defined as an IHC score of 3 or an IHC score of 2 with a FISH HER2/CEP17 score ≥2. Overall, the HER2/*ERBB2* concordance rate was 73.8% (59/80). The HER2/*ERBB2* positive percentage agreement among patients who were HER2‐positive was 63.3% (31/49), while the HER2/*ERBB2* negative percentage agreement among patients who were HER2‐negative was 90.3% (28/31). Furthermore, the HER2/*ERBB2*‐positive percentage agreements among patients who were IHC score of 3 was 67.6% (25/37). The HER2/*ERBB2*‐positive percentage agreements among patients who were IHC score of 2 with a FISH HER2/CEP17 score ≥2 was 50% (6/12). The HER2/*ERBB2*‐positive percentage agreement was higher in patients with an IHC score of 3 than in patients with an IHC score of 2 with a FISH HER2/CEP17 score ≥2.

## 4 DISCUSSION

**FIGURE 7:**
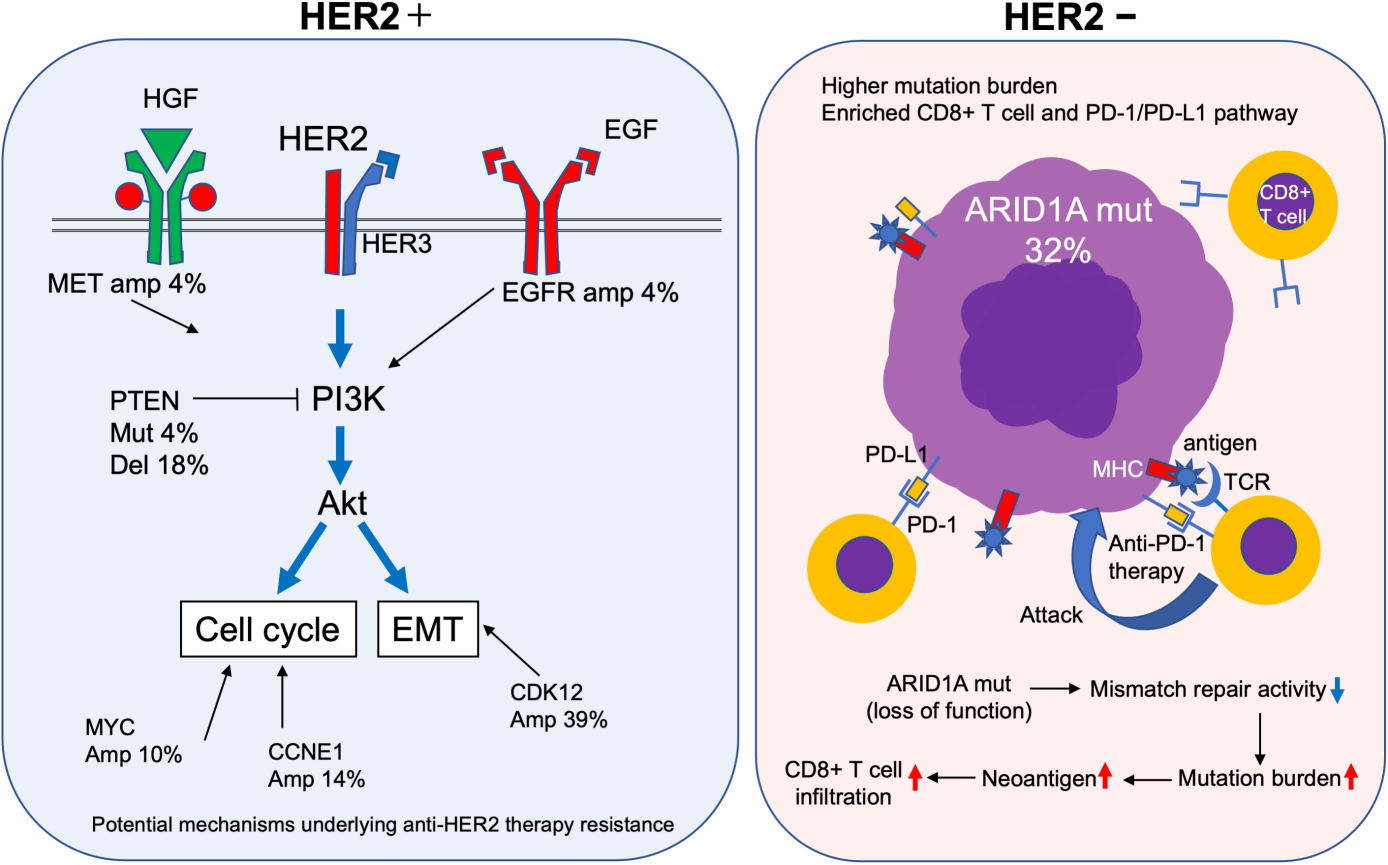
Overall summary of genomic characteristics of HER2‐positive and negative gastric cancer patients. Percentages around the gene symbol represent the frequency of gene alterations. Amp, amplification; Del, deletion; Mut, mutation.

Several elegant studies have described the genomic profile differences between HER2‐positive and HER2‐negative gastric cancer cases, suggesting the potential mechanism underlying trastuzumab resistance in these patients.^17^, ^18^, ^19^ However, most were small‐size retrospective studies and how the genomic differences potentially affect tumor progression in HER2‐positive and negative gastric cancer remains unclear. Using the CANCERPLEX‐JP 435‐gene panel, we profiled the genomic landscape of 49 HER2‐positive and 31 HER2‐negative gastric cancer patients who were enrolled in a prospective phase II trial. Specifically, we identified the enriched gene alterations and pathways based on patient HER2 status and examined the potential influences of the genomic differences on tumor progression and treatment responses (Figure 7).

In HER2‐positive tumors, we first focused on two enriched gene alterations: *PTEN* mutations and *PTEN* deletions. The *PTEN* mutations were p.T319fs, a frameshift mutation, and p.Q245_P248del, a novel nucleotide deletion, that were observed in two patients. Both can lead to the loss of PTEN function and subsequent activation of the PI3K/Akt pathway. Moreover, we observed *PTEN* deletions as CNVs in nine patients. *PTEN* deletions lead to low *PTEN* gene expression levels. Numerous studies have shown that *PTEN*‐negative tumors are unresponsive to trastuzumab, including breast and gastric cancers.^8^, ^9^, ^18^, ^20^ Thus, the observed *PTEN* mutations and deletions may be the potential mechanism underlying trastuzumab resistance in HER2‐positive gastric cancer.

Next, we found a significantly higher number of gene amplifications in HER2‐positive patients than in HER2‐negative patients, suggesting that gene amplification may be involved in acquired resistance to anti‐HER2 therapy. We focused on the enriched amplifications of *CDK12*, *MET*, and *EGFR* in HER2‐positive patients. Both *ERBB2* and *CDK12* are located at chromosome 17q12, which is a highly amplified region in gastric cancer.^21^ Consistent with several previous reports,^21^, ^22^, ^23^ our results suggested that *ERBB2* and *CDK12* were co‐amplified in 39% of HER2‐positive patients, as shown in Figure 2A. Interestingly, recent in vitro experiments showed that *CDK12* drove trastuzumab resistance and treatment with a *CDK12* inhibitor enhanced the sensitivity to anti‐HER2 therapy through the PI3K/Akt and Wnt pathways in breast cancer.^24^, ^25^ Thus, co‐amplification of *CDK12* and *ERBB2* may be a potential mechanism underlying trastuzumab resistance in gastric cancer. Furthermore, *MET* and *EGFR* are well‐known oncogenes that activate the PI3K/Akt pathway in breast cancer, so trastuzumab cannot completely mitigate this signaling.^26^, ^27^ These gene amplifications may also potentially affect the response to anti‐HER2 therapy in gastric cancer.

In HER2‐negative patients, we focused on the *ARID1A* mutations, which were the most enriched and the second most common after *TP53* mutations. Consistent with a previous report,^28^ the majority of *ARID1A* mutations in our study were inactivating mutations that can lead to the loss of *ARID1A* expression. Interestingly, ARID1A interacts with mismatch repair protein MSH2, and *ARID1A* deficiency could increase the mutation load across multiple human cancer types.^29^ Moreover, an *ARID1A*‐deficient ovarian cancer mice model displayed an increased mutation load, elevated numbers of tumor‐infiltrating lymphocytes, and higher PD‐L1 expression levels.^29^ These data indicate that HER2‐negative tumors with *ARID1A* mutation may be a “hot tumor” and encouraged us to further explore the associations between local immune environments and HER2 status in gastric cancer. Although only two tumors with *ARID1A* mutation were found in HER2‐positive patients (2/49), there were no obvious differences in mutation numbers and TMB between HER2‐positive tumors with *ARID1A* mutation and HER2‐negative tumors with *ARID1A* mutation. Therefore, the immunogenicity in HER2‐positive tumors with *ARID1A* mutation may be similar to that in HER2‐negative tumors with *ARID1A* mutation.

Pathway analyses showed that several immune‐related pathways were significantly associated with HER2‐negative gastric cancer, including PD‐L1 expression and the PD‐1 immune checkpoint pathway. Furthermore, compared with that in HER2‐positive patients, the TMB value calculated by the targeted NGS panel tended to be higher in HER2‐negative patients, particularly in patients with *ARID1A* mutation. In addition, TCGA whole exome sequencing dataset analyses showed that *ERBB2*‐nonamplified gastric cancer cases harbored higher mutation numbers, *CD8A* expression levels, and CYT scores. All these findings supported our hypothesis that HER2‐negative gastric cancer is a relatively “hot tumor,” while HER2‐positive gastric cancer is a relatively “cold tumor.” According to our hypothesis, patients with HER2‐positive gastric cancer may not benefit from immune checkpoint inhibitors. However, trastuzumab has been shown to activate both innate and adaptive immune responses through antibody‐dependent cellular cytotoxicity (ADCC)^30^ and antibody‐dependent cellular phagocytosis (ADCP).^31^ This is believed to be a reason why patients with HER2‐positive gastric cancer have better responses to immune checkpoint inhibitors when combined with trastuzumab therapy.^32^

The concordance rate of HER2 status and *ERBB2* amplification was 73.8% in this study, similar to the concordance rates in several previous reports.^33^, ^34^ Intratumor heterogeneity possibly induces the differences between HER2 status and *ERBB2* amplification. This study has two limitations. First, clinical data will only be available when the clinical analysis of the parent study is completed. Second, no in vivo or in vitro experiments have been conducted to further support our hypothesis. The results of this study should be validated in the future using genomic analysis that combines the clinical and experimental data.

It is well known that alcohol consumption is a risk factor for gastric cancer. Gastric ADH (alcohol dehydrogenase) is responsible for the majority of ethanol metabolism in human gastric cells and forms a metabolic barrier against orally administered alcohol.^35^, ^36^, ^37^ Therefore, gastric ADH may be involved in carcinogenesis, and it would be interesting to determine the role of ADH in gastric cancer. However, ADH is not included in the cancer gene panel used in this study. The association between gastric ADH and HER2 status should be investigated in the future.

In conclusion, here we showed the comprehensive genomic differences between HER2‐positive and HER2‐negative gastric cancer patients from a prospective trial. Several gene alterations in the HER2 pathway may be the potential mechanism underlying trastuzumab resistance. Moreover, relative to HER2‐positive gastric cancer, HER2‐negative gastric cancer cases with *ARID1A* mutation may be hot tumors that are responsive to immune checkpoint inhibitors.

## AUTHOR CONTRIBUTIONS

**Qingjiang Hu:** Conceptualization (lead); data curation (lead); formal analysis (lead); funding acquisition (lead); investigation (lead); methodology (lead); project administration (lead); resources (lead); software (lead); validation (lead); visualization (lead); writing – original draft (lead); writing – review and editing (lead). **Eiji Oki:** Data curation (equal); funding acquisition (equal); project administration (equal); supervision (equal). **Teppei Yamada:** Supervision (equal). **Tomomi Kashiwada:** Resources (equal). **Hideto Sonoda:** Resources (equal). **Masato Kataoka:** Resources (equal). **Hirofumi Kawanaka:** Resources (equal). **Yasushi Tsuji:** Resources (equal). **Akitaka Makiyama:** Resources (equal). **Yuichiro Nakashima:** Project administration (equal); resources (equal). **Mitsuhiko Ota:** Resources (equal); supervision (equal). **Yasue Kimura:** Resources (equal); supervision (equal). **Tomoharu Yoshizumi:** Supervision (equal).

## CONFLICT OF INTEREST STATEMENT

Eiji Oki reports research funding from Guardant Health, Inc. and reports honoraria from Ono Pharm., Takeda Pharm., Bayer, Chugai Pharm, Taiho Pharm., Eli Lilly Japan, and Bristol‐Myers Squibb. Yasue Kimura belonged to an endowed chair funded by Denka Company Limited from June 2020 to March 2023.

## ETHICAL APPROVAL STATEMENT

This study was approved by Kyushu University Certified Institutional Review Board for Clinical Trials. Informed consent was obtained from all participants.

## Supporting information


Figure S1.

Table S1.

Table S2.

Table S3.

## Data Availability

The data that support the findings of this study are available from the corresponding author upon reasonable request.
