# Supplementary figures and images for: Genomic characterization between HER2‐positive and negative gastric cancer patients in a prospective trial
Source: Cancer Med. 2023 Jun 16;12(15):16649–60. doi: 10.1002/cam4.6269 (PMC10469643; doi:10.1002/cam4.6269)

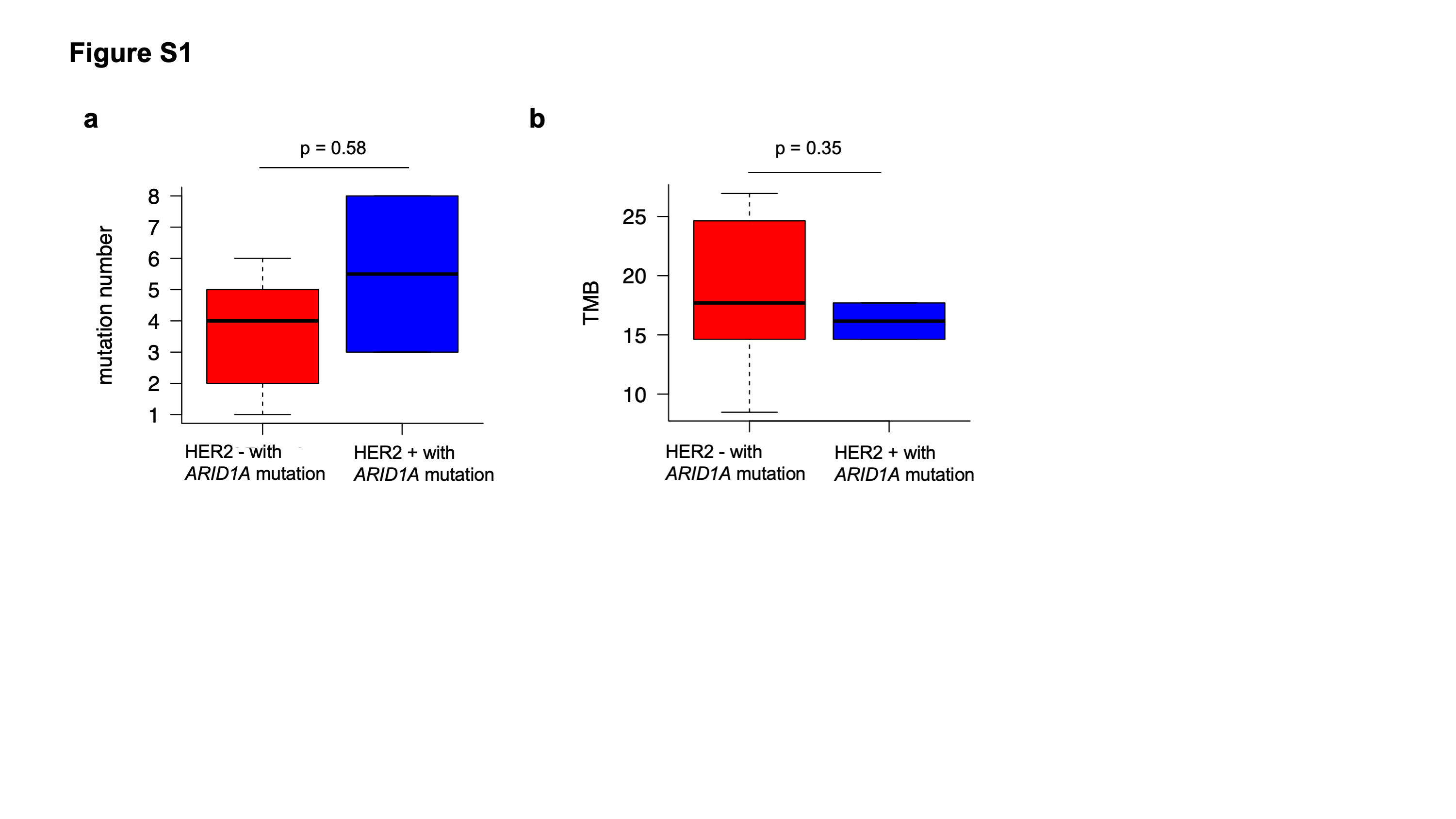

Supplement: Supplementary file 1 — Figure S1. [file CAM4-12-16649-s003.tiff]
