# Supplementary material for: Genomic characterization between HER2‐positive and negative gastric cancer patients in a prospective trial
Source: Cancer Med. 2023 Jun 16;12(15):16649–60. doi: 10.1002/cam4.6269 (PMC10469643; doi:10.1002/cam4.6269)
Supplement: Supplementary file 3 — Table S2. [file CAM4-12-16649-s001.docx]

**Table S2.** Background data of this study (TROX-A1 trial, n = 80).

|  | | HER2 | | Total |
| --- | --- | --- | --- | --- |
|  |  | Positive (n = 49) | Negative (n = 31) | n = 80 |
| Age, median (range) | | 68 (34-79) | 70 (52-86) | 69 (34-86) |
| Gender | Male (%) | 34 (69.4) | 25 (80.6) | 59 (73.75) |
|  | Female (%) | 15 (30.6) | 6 (19.4) | 21 (26.25) |
