# Supplementary material for: Genomic characterization between HER2‐positive and negative gastric cancer patients in a prospective trial
Source: Cancer Med. 2023 Jun 16;12(15):16649–60. doi: 10.1002/cam4.6269 (PMC10469643; doi:10.1002/cam4.6269)
Supplement: Supplementary file 4 — Table S3. [file CAM4-12-16649-s002.docx]

**Table S3.** Concordance between HER2 status determined by traditional methods and *ERBB2* amplification measured by NGS in this study (n = 80).

| HER2  (IHC and FISH) | *ERBB2* (CANCERPLEX-JP) | | Concordance rate |
| --- | --- | --- | --- |
|  | amplified | nonamplified |  |
| Positive (n = 49) | 31 (63.3%) | 18 (36.7%) | 73.8% |
| Negative (n = 31) | 3 (9.7%) | 28 (90.3%) |  |
| HER2 (IHC 3+) | | | |
| Positive (n = 37) | 25 (67.6%) | 12 (32.4%) | 77.9% |
| HER2 (IHC 2+ and FISH HER2/CEP17 score ≥ 2) | | | |
| Positive (n = 12) | 6 (50%) | 6 (50%) | 79.1% |
